# Editorial: Cell signalling in heart development, disease and regeneration

**DOI:** 10.3389/fphys.2023.1188406

**Published:** 2023-03-29

**Authors:** Carlos García-Padilla, Houria Daimi, Diego Franco, Estefanía Lozano-Velasco

**Affiliations:** ^1^ Cardiovascular Development Group, Department of Experimental Biology, University of Jaén, Jaén, Spain; ^2^ Department of Human Anatomy and Embryology, Faculty of Medicine, Institute of Molecular PAthology Biomarkers, University of Extremadura, Badajoz, Spain; ^3^ Biochemistry and Molecular Biology Laboratory, Faculty of Pharmacy, University of Monastir, Monastir, Tunisia; ^4^ Fundación Medina, Granada, Spain

**Keywords:** cardiac development, cell signalling, molecular pathways, transcription factors, non-coding RNAs, cardiac disease, regeneration

Heart development is a highly dynamic process tightly regulated by distinct cellular and molecular events during embryogenesis. This developmental process comprises the generation and coordination of multiple cell lineages, including cardiomyocytes, endothelial cells, epicardial cells and neural crest cells, establishing an intricate crosstalk signalling that promotes proliferation, differentiation, growth, cell survival and migration. Disturbances during cardiogenesis lead to distinct pathological phenotypes. Cardiovascular diseases, which affect millions of people, are one of the major causes of morbidity and mortality worldwide, including therein stroke heart, myocardial infarction or septic and/or dilated cardiomyopathy. The capacity for cardiac regeneration is lost in adult mammals, including humans, yet embryonic and early postnatal mammals display an extensive ability to restore cardiac injury through cardiomyocyte proliferation. The molecular mechanisms governing cardiac regeneration are poorly understood and represent an important milestone for current scientific research. Several reports revealed the contribution of distinct transcriptional and post-transcriptional molecular mechanisms in cardiac regeneration, pinpointing the importance of both the coding and non-coding genome. Furthermore, interactions between different cardiac cell populations are essential to improve cardiac function after cardiac injury, revealing that cardiomyocyte proliferation and therefore regeneration require signals from surrounding cells such as epicardial, cardiac fibroblast and endocardial cells. Within this Research Topic, four distinct papers have been gathered on different aspects of cardiac regeneration and the interplay between specific cardiac cell types.

In this Research Topic, Carta-Bergaz et al. have revealed a promising cellular therapy against ventricular tachycardias by limiting the epicardial arrhythmogenesis throughout the administration of cardiosphere-derived cells (CDCs). CDCs are considered an heterogeneous population of resident cardiac stem cells with the capacity to differentiate into distinct cardiac cell types, i.e., cardiomyocytes, endothelial cells or smooth muscle cells. The therapeutic use of CDCs has been widely explored, demonstrating their potential to promote cardiac regeneration and to improve cardiac function after myocardial infarction in humans and mice. However, the role of CDCs in ventricular tachycardias has been poorly analysed. Using the pig as experimental model, Carta-Bergaz et al. demonstrated that intrapericardial injection of CDCs after MI reduce drastically the formation of epicardial, but not the endocardial or myocardial scar, reducing the arrhythmogenic substrate. In addition, such a decreased electrical remodelling in the scar border zone increased epicardial electrical homogeneity by Cx43 upregulation. Furthermore, the authors show reduced fibrosis and increased cardiomyocyte viability in close apposition to the epicardial layer, suggesting that CDCs administration activates regenerative pathways from epicardium to myocardium.

Using a distinct experimental animal model, Francis et al. analysed the impact of oestrogens withdrawal in cardiomyocyte Ca^2+^ handling homeostasis. In previous studies, the authors have demonstrated that the long-term absence of ovarian hormones results in detrimental changes to cardiomyocyte Ca^2+^ handling homeostasis. In particular, a pro-arrhythmic phenotype characterized by asynchronous excitation-contraction (EC) coupling was identified. Furthermore, 17β-oestradiol administration restored proper Ca^2+^ handling, supporting the notion that circulating oestrogens have a cardiac protective role. To address the role of oestrogen-related proteins in cardiomyocyte Ca^2+^ handling, the authors forced activation of GPER, G-protein coupled oestrogen receptor 1, using a GPER agonist G-1 on cardiomyocytes from ovariectomised (OVx) guinea pig hearts. Curiously, OVx hearts showed increased protein levels of GPER compared to controls, suggesting that in lower oestrogens conditions, cardiomyocytes enhanced expression of this receptor with the aim of maximizing circulating estrogen uptake. Furthermore, the authors demonstrated that activation of GPER reduced Ca^2+^ transient (CaT) amplitude and Ca^2+^ spark and waves in OVx cardiomyocytes which represents the capacity of GPER to exert a protective role in stress-induced conditions such as in cardiomyocytes from females with low circulating oestrogens. In addition, GPER activation results in limited abnormal membrane depolarizations.

Yan et al. performed an exhaustive RNA sequencing time-series to analyse the cardiac gene expression pattern during cecal ligation and puncture (CLP)-induced sepsis in mice. A total of 5,607 genes were differently expressed in CLP-induced septic myocardium at different time points. GO ontology and KEGG pathways analyses revealed that transcriptomic profiles were associated with several processes and cellular signalling pathways involved in sepsis response, such as cell adhesion, immune system and inflammatory response. Furthermore, gene co-expression networks identified Pik3r1 and Pik3r5, two regulatory subunits of PI3K, as pivotal genes in the CLP process, suggesting a critical role of PI3K/AKT signalling in development and progression of cardiomyopathic sepsis. In addition, both isoforms displayed a stronger expression in septic myocardium. To address the importance of PI3K/AKT signalling in this process, the authors performed a loss of function assay of PI3Kγ, a mediator protein on triggering PI3K/AKT pathway, significantly ameliorating CLP-induced cardiac inflammation and NF-κB activation. Furthermore, Yan et al. analysed the impact of septic cardiomyopathy on cardiac homeostasis. CLP-treated mice exhibited a CLP time-dependently decreased myocardial contractile function and promoted myocardial inflammation accompanied by increased expression of IL-1β, IL-6, and TNF-α.

Finally, Althali and Hentges described a state-of-the-art clarifying study on the genetics that governs non-syndromic Tetralogy of Fallot (TOF), pinpointing the importance of genetics Research Topic on several transcription factors as potential cause and/or consequence of TOF development and pathology. The authors highlighted that NOTCH1 locus, followed by FLT4 and NKX 2.5, are the most common genes of genetic variants observed in non-syndromic TOF patients. Curiously, 7% of TOF cases are caused by mutations in these genes. In addition, the authors emphasize the impact of incorrect interaction between several cardiac transcriptional factors involved in heart morphogenesis as a potential cause of TOF.

In sum, these four papers published in this Research Topic highlight the potential therapeutic use of CDCs pericardial injection, oestrogens hormone administration and PI3Kγ depletion in arrhythmogenesis, Ca^2+^ handling homeostasis and cardiomyopathic sepsis, respectively. Thus, these four papers increase our understanding of the complex relationship between cardiac cell populations and/or genetic underlying in several cardiac diseases.

